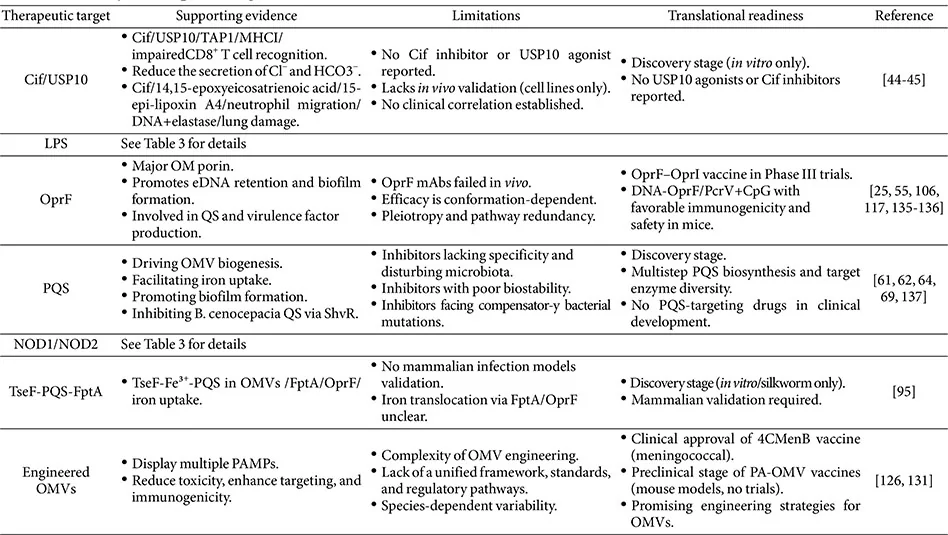# Targeting Outer Membrane Vesicles of *Pseudomonas aeruginosa*: From Pathogenesis to Therapeutics

**DOI:** 10.4014/jmb.2605.05019

**Published:** 2026-09-02

**Authors:** Shuaiyuan Liang, Nan Gao, Fan Fei, Xiaoqin Jin, Jiaxin Liu, Xiuwei Zhang, Xingran Du

**Affiliations:** 1 Department of Respiratory and Critical Care Medicine, The Affiliated Jiangning Hospital with Nanjing Medical University, Nanjing 211100, P. R. China; 2 Department of Respiratory and Critical Care Medicine, The Second Affiliated Hospital of Nanjing Medical University, Nanjing 210011, P. R. China

**Keywords:** Outer membrane vesicles, *Pseudomonas aeruginosa*, Pathogens, Potential targets, Engineered PA-OMVs

## Abstract

Multidrug-resistant *Pseudomonas aeruginosa* (*P. aeruginosa*) causes a variety of illnesses and has become a major challenge in clinical settings. Outer membrane vesicles (OMVs) have attracted extensive research interest in recent years and are considered a promising platform for managing *P. aeruginosa* infections. Therefore, it is essential to comprehend the complex mechanisms and possible targets that underlie the interactions between pathogens and OMVs. In this review, we summarize the key components of *P. aeruginosa* OMVs (PA-OMVs), elucidate their pathogenicity mechanisms, and discuss their potential therapeutic targets. Finally, we provide an overview of recent advances in PA-OMV research.

## Introduction

**Fig. 1:**
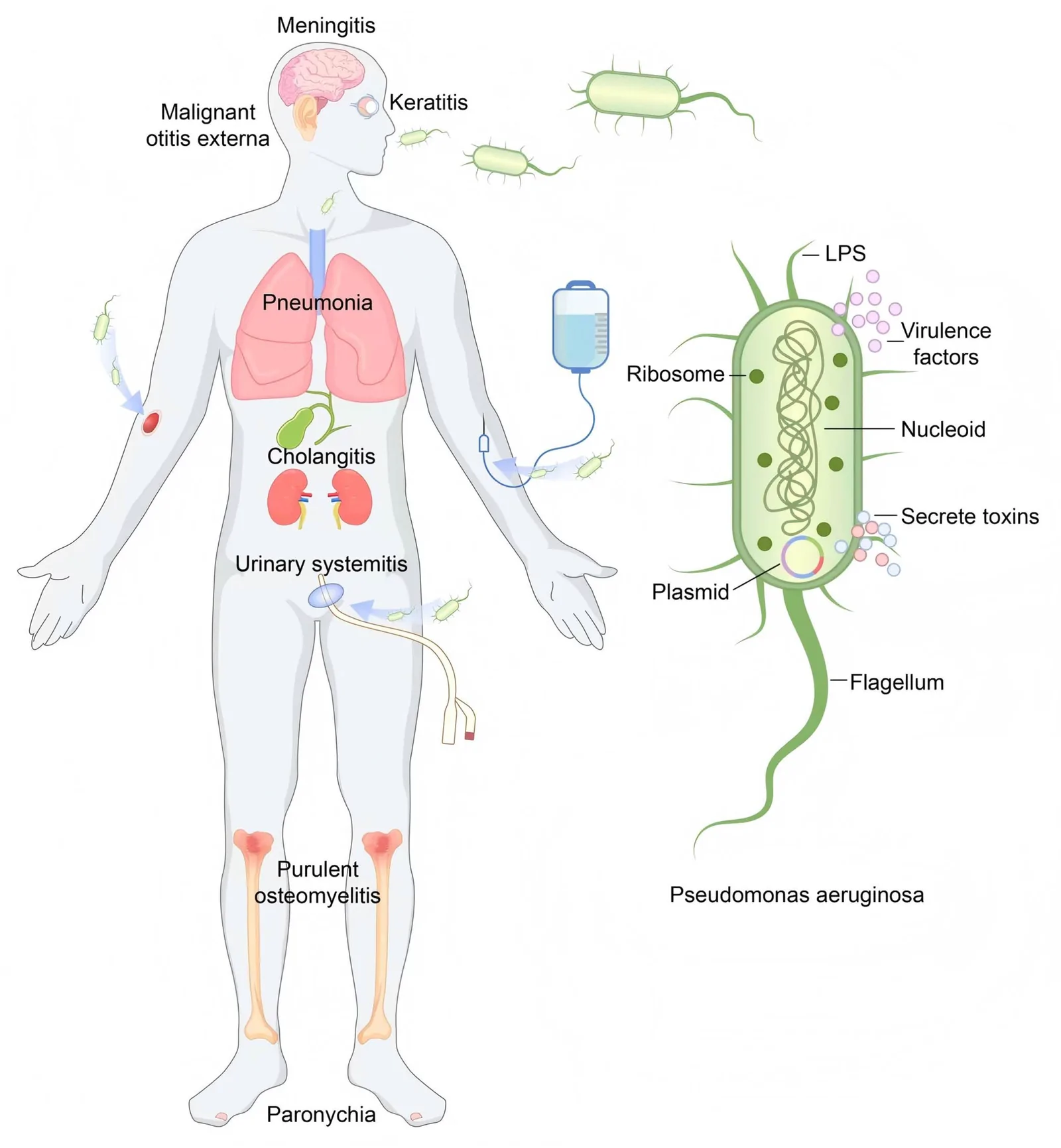
*P. aeruginosa*: Routes of entry and systemic infections. *P. aeruginosa* is widespread and enters the body through broken skin, medical devices, or inhalation, causing severe infections in various organs, including lungs, bones, ears, and brain.

The multidrug-resistant (MDR) Gram-negative opportunistic pathogen *Pseudomonas aeruginosa* (*P. aeruginosa*) inhabits diverse environments, including soil, water, plants, human skin, and oral mucosa. It frequently colonizes patients with cystic fibrosis, sepsis, bronchiectasis, and ventilator-associated pneumonia [1]. In immunocompromised hosts, *P. aeruginosa* can establish acute or chronic infections, leading to life-threatening conditions with mortality rates as high as 40% (Fig. 1) [2]. Moreover, *P. aeruginosa* is among the leading causes of hospital-acquired infections and is classified as one of the ESKAPE pathogens (*Enterococcus faecalis*, *Staphylococcus aureus*, *Klebsiella pneumoniae*, *Acinetobacter baumannii*, *P. aeruginosa*, and *Enterobacteriaceae* spp.) [3]. The World Health Organization has identified *P. aeruginosa* as a significant global threat [4].

Over the past decade, the prevalence of antimicrobial resistance in *P. aeruginosa* clinical isolates has increased markedly worldwide. The 2024 GRAM-Lancet analysis reported that 4.71 million human deaths were associated with bacterial antimicrobial resistance in 2021, with 1.14 million directly attributable to resistant infections [5]. Consequently, the number of effective antimicrobials against *P. aeruginosa* is decreasing. It is predicted that by 2050, no effective antibiotics may remain unless new therapeutics are developed [6]. Therefore, identifying novel therapeutic targets and developing new drugs against *P. aeruginosa* offers a promising strategy to improve treatment efficacy.

In recent years, alternatives to conventional antibiotics—including metal ions, antimicrobial peptides, and bacteriophages—have been explored and shown promise. However, these alternatives face limitations such as cytotoxicity, potential for resistance, limited clinical efficacy, and poor storage stability, which significantly hinder their clinical translation [7-9]. In this context, outer membrane vesicles (OMVs) have emerged as a highly attractive platform for future applications. Notably, an OMV-based vaccine against *Neisseria meningitidis* is already commercially available, underscoring the substantial potential of OMVs in biomedicine [10].

Previous research has demonstrated that all cells can produce extracellular vesicles as part of their normal growth [11]. In bacteria, extracellular vesicles were first described in 1965 [12]. OMVs are precisely defined as vesicles that originate from Gram-negative bacteria, such as *Acinetobacter baumannii* and *P. aeruginosa* [3]. OMVs are spherical, bilayered nanostructures (20–400 nm) [14]. OMVs are currently understood to arise primarily through membrane blebbing and explosive cell lysis [15]. Studies indicate that OMVs generated via various biogenesis pathways differ in functional enrichment and proteomic profiles, which can either promote bacterial survival or contribute to host infection [16].

To effectively harness OMVs, expanding our knowledge of the composition and complex biogenesis of *P. aeruginosa* OMVs (PA-OMVs) is crucial. In this review, we summarize the components of PA-OMVs, discuss recent advances in potential pathogenic targets associated with PA-OMVs, and explore the development and application prospects of engineered PA-OMVs.

## Components of OMVs and Major Porin Family Constituents

**Fig. 2:**
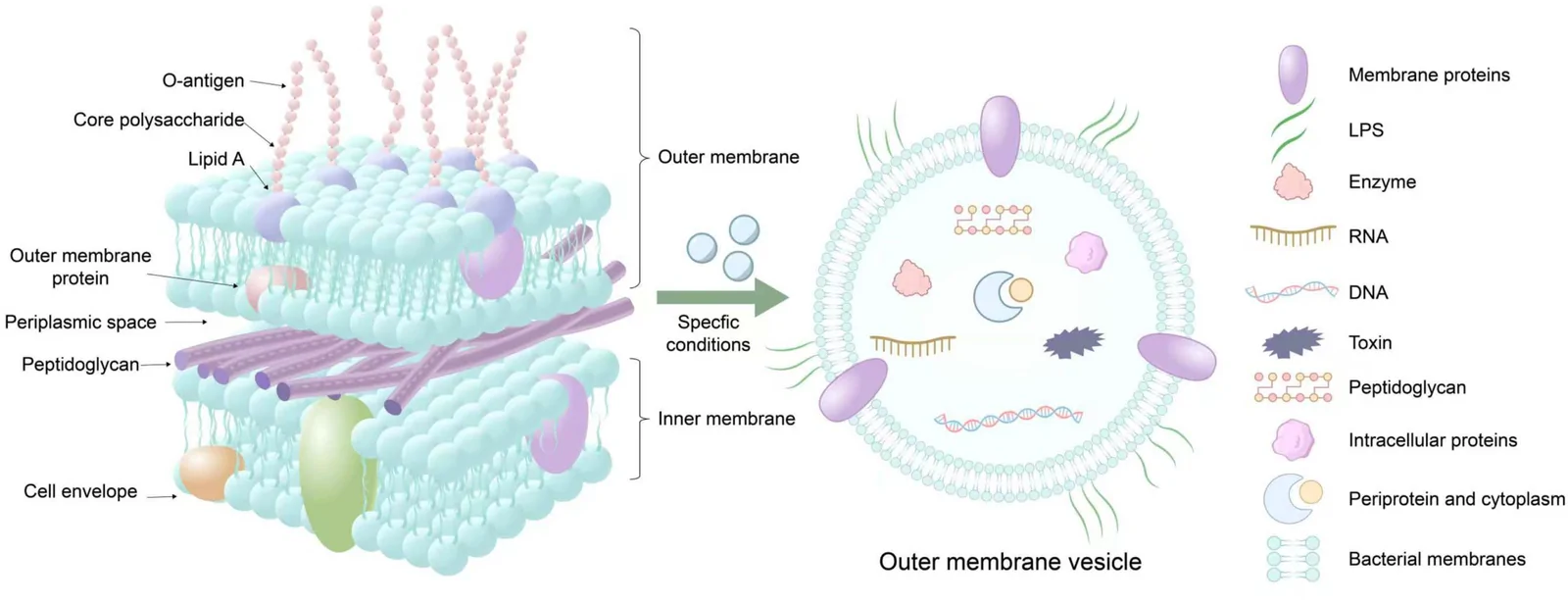
The formation and composition of PA-OMVs. The components of OMVs primarily include elements of the outer membrane and periplasmic space, such as LPS, outer membrane proteins, peptidoglycan, enzymes, and DNA molecules.

OMVs have been extensively studied, and their composition is complex. Notably, while the components of OMVs are generally similar to those of the bacterial outer membrane, their relative proportions (*e.g.*, phospholipids and fatty acids) may differ [17]. Under specific conditions—such as membrane stress or exposure to environmental stressors—the outer membrane of *P. aeruginosa* can undergo localized expansion, leading to OMV formation. The components of OMVs primarily include elements of the outer membrane and periplasmic space, such as LPS, outer membrane proteins, peptidoglycan, enzymes, and DNA [18]. The specific composition of PA-OMVs is shown in Fig. 2.

*P. aeruginosa* contains numerous proteins, including lipoproteins, transport proteins, secretion system-associated proteins, and porins. Porins, the most extensively studied and abundant proteins in the outer membrane, are named for their pore-like morphology and serve as channels [19]. These include non-specific porins (OprF), specific porins (OprB, OprD, OprE, OprO, and OprP), gating porins (OprC and OprH), and efflux-related porins (OprM, OprN, and OprJ) [20]. *P. aeruginosa* has been shown to have twelve efflux pumps. MexAB-OprM, MexCD-OprJ, MexEF-OprN, and MexXY-OprM are four of these efflux pumps that make a substantial contribution to *P. aeruginosa*'s resistance to many antibiotics. The main adopted substances and physiological functions of *P. aeruginosa* porins are summarized in Table 1.

## Pathogenic Role and Potential Targets of OMVs in *P. aeruginosa*

**Fig. 3:**
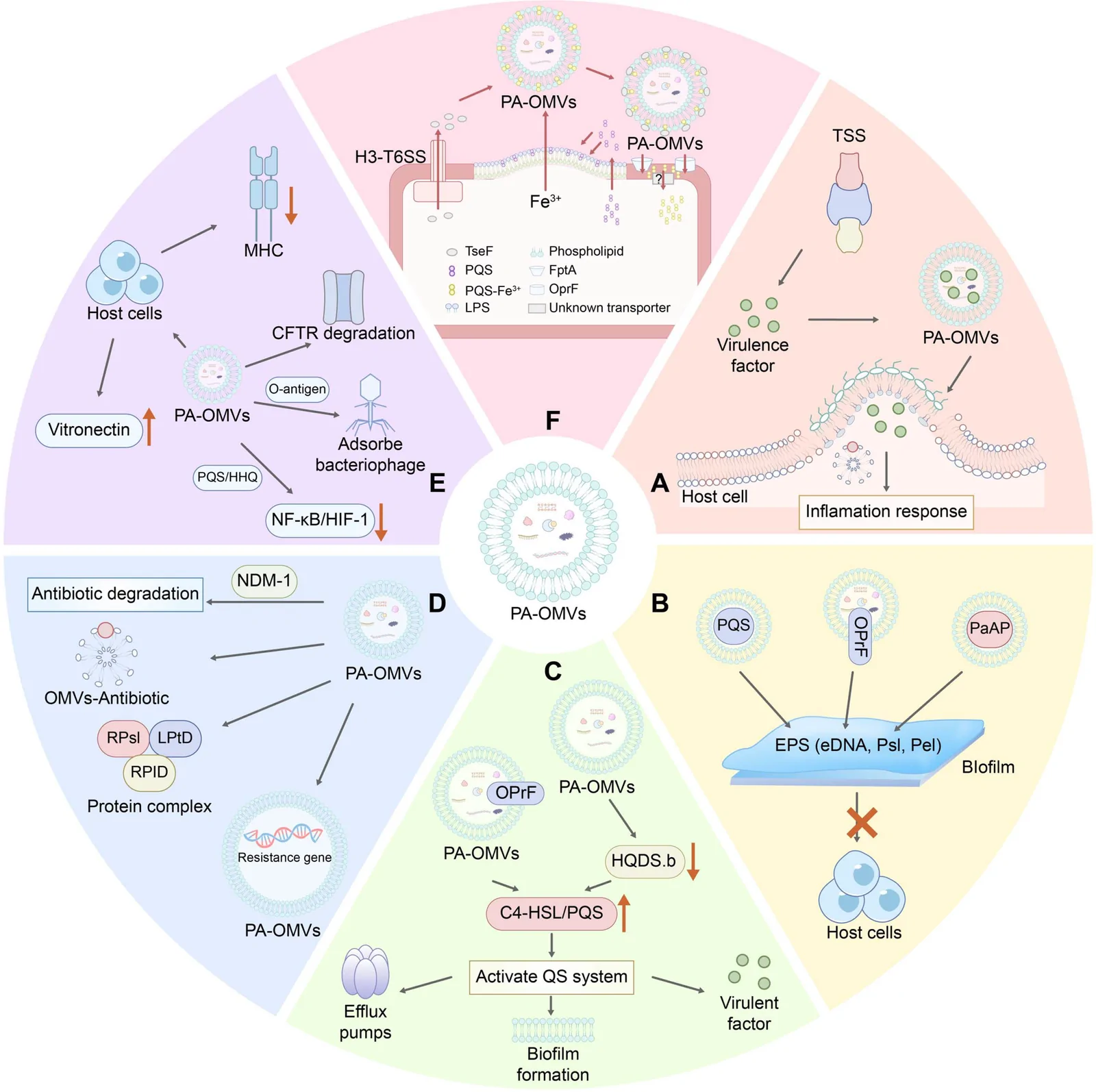
Diagram of the main pathogenic mechanisms of PA-OMVs. (**A**) PA-OMVs can selectively encapsulate virulence factors and directly transport virulence factors to host cells by means of membrane fusion. (**B**) Various components of PA-OMVs promote biofilm formation. (**C**) Some of the mechanisms associated with the PA-OMVs promote the production of self-inducing agents, leading to the activation of the QS system and facilitating the expression of efflux pump expression, biofilm formation, production of virulence factors, etc. (**D**) PA-OMVs can promote antibiotic resistance in a variety of ways, including degradation of antibiotics, binding of antibiotics, formation of complexes between ribosomal proteins and drug targets, and carriage of antibiotic resistance genes, etc. (**E**) In the regulation of host immunity, there are two main aspects: a) suppression of the immune system, through the degradation of CFTR molecules, adsorption of O-antigen to phage, inhibition of the NF-KB/HIF-1 signaling pathway, promoting the elevation of vitronectin and decreasing the level of MHC molecules, and so on. b) deregulated activation of the inflammatory signalling pathway. (**F**) TseF is secreted by H3-T6SS and then taken up by PA-OMVs containing PQS-Fe^3+^. TseF is recognised by the cell surface receptors FptA or OprF, which translocate iron into the cell by an as yet undefined mechanism.

Despite substantial research on the significance of PA-OMVs, a comprehensive evaluation of their pathogenic function and therapeutic targets is lacking. This section examines the diverse pathogenic roles and potential targets of OMVs, offering new research avenues for tackling antibiotic resistance. The main pathogenic mechanisms of PA-OMVs are illustrated in Fig. 3.

### Transmission of Virulence Factors and Horizontal Gene Transfer of Toxin Genes

Virulence factors are synthesized and secreted by bacterial secretion systems [40]. PA-OMVs function as highly effective delivery vehicles by merging with lipid rafts in the host plasma membrane [41]. This process facilitates the translocation of multiple virulence factors—including lipopolysaccharide (LPS), alkaline phosphatase, and Cif, an inhibitor of the host cystic fibrosis transmembrane conductance regulator (CFTR) [14, 42]—into the cytoplasm of airway epithelial cells through N-WASP-mediated actin pathways [43]. These mechanisms not only undermine the host's antimicrobial barrier function but also accelerate chronic infection by disturbing epithelial cell homeostasis.

Cif acts as an epoxide hydrolase, reducing Cl^-^ secretion by facilitating the lysosomal degradation of CFTR, thereby hindering pathogen clearance by mucosal cilia [44]. Moreover, Cif inhibits Ubiquitin-specific protease-10 (USP10), leading to aberrant polyubiquitination of Transporter associated with Antigen Processing 1 (TAP1). This alteration ultimately diminishes major histocompatibility complex (MHC) class I antigen presentation at the plasma membrane and obstructs CD^8+^ T cell-mediated pathogen clearance [45]. The antigen-presenting cells used in this mechanism study were EL-4 and MLE-K cells. This pathway enables immune evasion during co-infections involving *P. aeruginosa* and lung commensal flora such as *Haemophilus influenzae*. The discovery of activators targeting USP10 may offer a viable strategy for restoring host antigen presentation and reversing bacterial immunosuppression.

As a major virulence factor of *P. aeruginosa*, LPS is a typical pathogen-associated molecular pattern (PAMP) recognized by pattern recognition receptors (PRRs), which activates the NLRP3 inflammasome and causes tissue damage in both acute and chronic infections [46]. Mutations in LPS may lead to decreased virulence while allowing bacteria to acquire resistance to phage infections. Caffeine mitigated *P. aeruginosa*-induced lung injury by negatively modulating the cAMP/PKA/NF-κB axis, which in turn downregulated miR-301b expression (upregulated by the LPS-activated TLR4/MyD88/NF-κB pathway). This reduced hyperinflammation and maintained alveolar epithelial barrier integrity [47].

Virulence factor expression is precisely controlled by the quorum-sensing (QS) system. This system senses the population density by releasing self-inducers such as N-butanoyl-L-homoserine lactone (C4-HSL) and N-(3-oxododecanoyl)-L-homoserine lactone (3-oxo-C12-HSL), thereby adjusting the timing of virulence gene expression to counteract host immune responses [20].

Contemporary strategies for reducing the toxicity of virulence factors include the use of deoxycholate detergent and genetic modification of LPS [48]. These approaches aim to reduce endotoxicity and overall toxicity. In the future, a novel therapeutic strategy against *P. aeruginosa* infections may involve attenuating virulence by targeting the QS system.

### PA-OMV-Mediated Regulation of Biofilm Formation

Numerous bacteria inhabit their natural environments as aggregated or surface-attached colonies, also known as biofilms [49]. Biofilms consist of water, bacterial organisms, and various indigenous extracellular polymeric substances (EPS) [50]. EPS generated by *P. aeruginosa* comprises nucleic acids, extracellular polysaccharides, lipids, and proteins. Among these, three distinct extracellular polysaccharides—alginate, Pel, and Psl—have been recognized as pivotal factors in biofilm formation [51]. PA-OMVs regulate biofilm formation through three aspects: serving as structural components of EPS, acting as carriers of *Pseudomonas* quinolone signal (PQS) molecules, and exerting strain-specific effects.

First, PA-OMVs serve as integral structural constituents of the EPS. OMVs have been demonstrated to be a critical component of EPS [52]. Their absence leads to structural defects in biofilms, thereby restricting biofilm development [53]. OprF has been identified as the primary porin in OMVs [54], exhibiting nutrient-dependent characteristics that help retain extracellular DNA (eDNA) within the biofilm matrix, where OprF functions as a binding agent. Additionally, OprF promotes biofilm formation and adhesion in *P. aeruginosa*, thereby contributing to enhanced antibiotic resistance within biofilms [55]. Therefore, knocking down or inhibiting OprF may represent a therapeutic strategy.

Second, PA-OMVs function as carriers of PQS signaling molecules that modulate community interactions. Extensive research indicates that PA-OMVs can load and transport PQS molecules, significantly enhancing biofilm formation efficiency [25]. Furthermore, PA-OMVs efficiently transfer PQS to other bacteria, inhibiting their growth and thereby reducing microbial community diversity and abundance within biofilms. However, varying PQS-OMV doses can shift dominant bacterial species, likely due to differential sensitivities to OMVs [56].

Third, regarding strain specificity, a multivariate analysis using three distinct 96-well plates simultaneously evaluated the effects of multiple physicochemical parameters under various conditions. The analysis revealed a significant negative correlation between biofilm formation in LPS mutant strains and OMV biosynthesis [57]. *P. aeruginosa* strains expressing the *P. aeruginosa* peptidase (PaAP) help regulate protease activity within OMVs. This ultimately increases the Psl/biomass ratio in the early biofilm matrix, leading to biofilm structural remodeling that protects developing colonies from antimicrobial treatment [58].

### Interplay between QS Signaling and PA-OMV Biogenesis

The QS system is an intercellular communication mechanism that enables bacteria to coordinate collective behaviors in response to population density by secreting self-inducers [59]. Among its four subsystems—Las (long-chain acyl-homoserine lactone synthase), Rhl (rhamnolipid biosynthesis), PQS, and IQS (integrated quorum sensing)—PQS plays a pivotal role in OMV biogenesis, in addition to its canonical function as a signaling molecule [60]. Numerous physiological processes and collective behaviors, such as biofilm formation [61], iron acquisition [62], and efflux pump expression [63], are regulated by the QS system.

On the one hand, the QS system (particularly PQS) drives OMV production. PQS promotes OMV production through a bilayer coupling model. Due to its hydrophobic characteristic, PQS preferentially incorporates into the outer leaflet of the outer membrane, resulting in an imbalance in membrane tension that alters membrane curvature and triggers vesicle release [64]. For PQS to be incorporated into the lipid bilayer, it must navigate a designated translocation pathway and ultimately integrate into OMVs [65]. Without PQS, OMV output is markedly diminished [25]. It is essential to recognize that OMV production is directly correlated with PQS output rather than total synthesis [66]. Therefore, targeting the PQS output pathway with inhibitors offers a novel anti-infection strategy. OprF significantly affects OMV synthesis by modulating PQS formation, regardless of PQS levels [25]. Also, OprF emerges as a viable target for intervention.

On the other hand, OMVs can reciprocally modulate the QS system. First, both OMVs and sublethal doses of polymyxins were observed to downregulate QS-related gene transcription. When combined, OMVs and polymyxin B exhibited a synergistic impact, considerably decreasing the release of virulence factors, bacterial motility, and biofilm formation within the QS system of *P. aeruginosa* [67], highlighting the potential of OMV-antibiotic combination therapy. Second, OMVs can associate with 3-hydroxy-4(1H)-quinolone 2,4-dioxygenase from Streptomyces bingchenggensis (HQDS.b.), which degrades PQS and thereby attenuates quorum sensing, reducing pathogenicity by inhibiting biofilm formation and virulence factor production [68]. The HQDS.b.-OMV complex may serve as a prospective tool for anti-infection therapy. Third, PQS contained within PA-OMVs suppresses the QS system of *Burkholderia cenocepacia* by specifically targeting the ShvR protein [69], leading to diminished biofilm formation, reduced virulence factor production, and suppression of the overall QS system.

### Promoting the Emergence of Antibiotic Resistance

The complexity and dynamic evolution of *P. aeruginosa* resistance mechanisms have significantly diminished the effectiveness of current antibiotics, resulting in fewer treatment options for clinicians.

OMVs, functioning as natural biological nanoparticles, can encapsulate a variety of cargoes, including antibiotic resistance genes [70]. They enable horizontal gene transfer (HGT) to recipient bacteria and promote both intra- and interspecies dissemination of resistance determinants. Notably, this form of horizontal gene transfer may operate independently of conventional mechanisms, including natural transformation, phage transduction, and type IV secretion systems [71]. Studies have shown that *E. coli* can directly transfer the NDM-1 protein to *P. aeruginosa* strains via OMVs, significantly enhancing the survival of *P. aeruginosa* under antibiotic pressure [72]. Some researchers have constructed a library of long-acting nitroxoline derivatives and identified ASN-1733 as a promising antibiotic candidate. It combats resistant bacterial infections by competitively inhibiting the NDM-1 enzyme [73]. A study using a *Drosophila* larvae model revealed that *Klebsiella pneumoniae* carbapenemase (KPC)-loaded OMVs not only hydrolyze imipenem *in vitro* and *in vivo*, but also induce carbapenem-resistant mutations in *P. aeruginosa* at low doses, thereby promoting the emergence of drug resistance [74]. Subsequent investigations have confirmed through both *in vivo* and *in vitro* experiments that chlorogenic acid exerts its anti-infective efficacy by targeting KPC-OMVs [75].

In parallel, PA-OMVs may promote the development of antibiotic resistance through mechanisms other than the transfer of resistance substances. For instance, OMVs can provide immediate protection to bacteria before they develop target modifications or mutations by serving as decoys that bind and sequester antibiotics and toxins [76]. PA-OMVs organize functional complexes by integrating various resistance-associated proteins. Cytoplasmic proteins enriched in OMVs, such as ribosomal proteins, form a synergistic network with outer membrane targets, including LptD/OstA and porins such as OprD/OprE [77]. This coordinated interaction effectively diminishes antibiotic entry into cells. Additionally, reduced OprD expression is directly correlated with increased carbapenem resistance [78]. Furthermore, OMVs carrying metallo-β-lactamases (MBLs) not only decrease local antibiotic concentrations through drug sequestration but also function as carriers for the global dissemination of resistance determinants such as NDM-1 [79]. Clinical observations demonstrate that the acidic microenvironment in the airways of individuals with chronic respiratory illnesses, such as bronchiectasis, can significantly enhance PA-OMV production. Mechanistic studies indicate that acidic stress increases the synthesis of 2-heptyl-4-quinolone (HHQ) in *P. aeruginosa* by modulating the PQS, thereby promoting infection [80]. Nonetheless, additional investigations utilizing in situ detection methods, including those involving physiological concentrations of OMVs and *in vivo* trials, are required to corroborate the underlying mechanisms.

### PA-OMV-Induced Inflammatory and Immunopathological Responses

PA-OMVs employ distinct immune evasion strategies at different growth stages. These include increasing alginate biosynthesis [81], downregulating virulence factor expression [82], and enhancing resistance to phagocytic clearance. For example, the PA-OMVs-associated virulence factor Cif weakens airway innate immunity by promoting the degradation of CFTR [44]. Also, PA-OMVs can bind to phage via LPS, especially O-antigen, thereby reducing phage efficacy against infected cells [83]. Another study discovered that LPS makes type II alveolar epithelial cells release vitronectin, conferring protection against complement-mediated killing [84]. It is also speculated that vitronectin can be used to detect *P. aeruginosa* infections. Lung macrophages (LM) are the first line of defense. However, PA-OMVs suppress the expression of MHC-associated molecules in lung macrophages [85]. PA-OMVs act as viral mimics, sequestering host antimicrobial factors and enabling bacterial evasion of the immune system during colonization [86]. In another study, the PQS and HHQ have been proven to compromise host defenses by suppressing the NF-κB and HIF-1 pathways [87].

In the absence of viable bacterial cells, PA-OMVs induce significant inflammatory responses [88]. The upregulation of inflammatory markers initiates the sequential release of chemokines (such as C-X-C motif chemokine ligand 1 and C-C motif chemokine ligand 2) and pro-inflammatory cytokines (such as IL-1β, TNF-α, IL-6, and IFN-γ), ultimately resulting in peribronchial infiltration and lung parenchyma inflammation. Concurrently, PA-OMVs induce mitochondrial dysfunction, activate intrinsic apoptotic pathways (including caspase-9), and promote mitochondrial DNA release—all of which synergistically exacerbate the inflammatory cascade [89]. PA-OMVs regulate macrophage metabolism via the TLR2/4-PI3K/Akt axis, promoting aerobic glycolysis to drive inflammation. By enhancing the proinflammatory state, glycolysis has been identified as a potential therapeutic target for bacterial-associated inflammatory diseases [90].

While activation of the inflammatory signaling pathway enhances host defense mechanisms against pathogens, dysregulated activation (particularly sustained NLRP3 inflammasome activity) leads to pathological overproduction of pro-inflammatory mediators, including IL-1β and IL-18 [46]. CprA-containing PA-OMVs prolong NLRP3 inflammasome activation and exacerbate cytokine storm syndrome through autophagy inhibition [95]. In contrast, OprC-deficient strains demonstrate significantly reduced macrophage pyroptosis and attenuated lung injury via suppression of both TLR2/4 signaling and inflammasome activation, improving survival rates in murine models [35]. The development of pharmacological inhibitors targeting NOD1/RIP2 or caspase-5/11 signaling pathways represents a promising therapeutic strategy for preventing aberrant inflammasome activation. Notably, the 5' formyl-methionine tRNA halves (5' tRNA-fMet halves) have been identified as a critical regulator that effectively suppresses OMV-induced IL-8 production and neutrophil infiltration [92].

### Disruption of Metal Ion Homeostasis

Iron is an essential cofactor for almost every biological function, playing a vital component in enzymatic reactions, regulatory proteins, and protein complexes involved in metabolism and signaling pathways [93]. PA-OMVs have been found to employ an additional, independent mechanism of iron competition.

The signaling molecule PQS functions as an autoinducer with a high affinity for iron [94], chelating Fe^3+^ and enhancing the expression of biosynthetic genes related to Fe^3+^ carriers. This study has elucidated a sophisticated iron uptake system involving the type VI secretion system (T6SS), PQS, and PA-OMVs. Type VI secretion system effector F (TseF) (encoded by gene PA2374) is released by the H3-T6SS and forms a complex with OMVs that are enriched with PQS and Fe^3+^. TseF facilitates the delivery of OMV-associated iron to bacterial cells by interacting with the Fe(III)-pyochelin receptor FptA and the porin OprF. Further analyses indicate that OprF may act as a receptor or channel protein for TseF, enabling the transmembrane transport of the Fe³+-PQS complex [95]. From a therapeutic strategy perspective, targeted disruption of TseF function or interference with the TseF-PQS-FptA complex could effectively suppress iron-dependent virulence expression in *P. aeruginosa*. Furthermore, the suppression of PA-OMV production or PQS packaging may restrict iron distribution throughout bacterial populations, presenting intriguing targets for the creation of novel antibacterial drugs.

In addition to their role in iron acquisition, PA-OMVs can directly act on sodium ion (Na^+^) channels in cardiomyocytes, delaying their inactivation. This leads to increased Na^+^ influx, which in turn promotes elevated intracellular Ca^2+^ levels, triggering arrhythmias and loss of contractility. In animal experiments, intravenous injection of purified PA-OMVs into mice successfully reproduced cardiac abnormalities and increased mortality. Neutralizing key bacterial antigens packaged within OMVs may represent a potential therapeutic strategy for treating ICU patients with *P. aeruginosa* infections [96].

## Engineered PA-OMVs for Therapeutic and Vaccine Applications

Although PA-OMVs exhibit diverse pathogenic mechanisms, they also exert certain protective effects on the host. First, OMVs derived from *P. aeruginosa* possess inherent immunogenicity due to their content of multiple antigens, such as OprF, OprI, and exotoxin A. These antigens can be processed and presented to T cells by antigen-presenting cells (APCs) via MHC molecules, thereby initiating an adaptive immune response. Simultaneously, cytokine signaling activates B cells, promoting antibody production [97]. Similar to other Gram-negative bacterial OMVs, PA-OMVs are recognized by PRRs. This recognition is mediated by PAMPs displayed on OMVs (such as LPS and peptidoglycan) [98]. The immunological features and vaccine relevance of major Gram-negative PAMPs are summarized in Table 2. Additionally, PA-OMVs may ameliorate lung I/R injury by regulating the balance of Tregs and Th17 cells through the Tim-3 and TLR4/NF-κB pathway [125]. Collectively, OMVs represent a promising vaccination platform. For instance, the OMV-based meningococcal vaccine 4CMenB is expected to provide 33% to 47% protection against gonorrhea [126]. PA-OMV vaccines have demonstrated protective effects in preclinical models. For example, immunization of mice with PA-OMVs formulated with aluminum hydroxide adjuvant reduced bacterial colonization, suppressed inflammatory cytokine production, and significantly improved survival rates during lethal pulmonary infection challenges [127]. Therefore, OMVs have considerable potential for future clinical application. Nonetheless, the intrinsic inflammatory properties of OMVs provide obstacles to their use as vaccine platforms.

Engineered OMVs have been developed via genetic editing, chemical modification, or synthetic biology techniques to enhance their properties, such as target specificity and drug-carrying capacity, or to impart novel functionalities. A primary objective is to diminish toxicity and adverse effects while enhancing the immunological response, which is especially vital for immunocompromised and debilitated patients.

### PA-OMVs Engineering: Strategies and Technological Innovations

A novel approach involves biomimetic cationic lipid hybrid vesicles (BCVs) integrated with the CRISPR/Cas9 plasmid delivery system. BCVs utilize the positive charge of lipids to improve targeting specificity while selectively cleaving bacterial DNA for efficient bactericidal and anti-biofilm effects [128]. This technique has exhibited superior safety and programmability in animal models, providing a tailored therapeutic approach for drug-resistant bacterial infections. Furthermore, detoxified synthetic OMVs (SyBVs) produced through enzymatic treatment and ultrasonic fragmentation markedly decrease inflammatory toxicity while preserving the capacity to elicit humoral and cellular immune responses, making them promising candidates for vaccine development, especially against viruses like SARS-CoV-2 [129]. An innovative method, nitrogen cavitation, mechanically disrupts cell membranes to generate double-layered membrane vesicles (DMVs). In a *P. aeruginosa* sepsis model, DMVs augmented both innate and adaptive immunity, and led to dramatically higher survival rates in mice [130]. This straightforward yet unique method has significant potential as a future technology for vaccine development.

### Groundbreaking Investigations on Multifunctional Composite Vesicles

Research on engineered OMVs has advanced from a singular component or function to multifunctional integrated composite vesicles. Hybridized membrane vesicles (HMVs) were created by fusing macrophage membrane vesicles (MMVs) with PA-OMVs, followed by conjugation with gold nanoparticles (AuNPs) and bacterial secreted toxins (SPs) to construct the multi-antigen vaccine, AuNP@HMV@SPs [131]. This vaccine not only diminished the inflammatory toxicity and hemolytic activity of OMVs but also achieved a 100% survival rate in a *P. aeruginosa* sepsis model, while considerably reducing infection severity in a pneumonia model. Additionally, studies have shown that Pf4-associated OMVs carry small RNAs capable of activating the TLR3 pathway and promoting IFN-β production, while also reducing macrophage hyperinflammatory responses to LPS [132]. This method offers an innovative tool to regulate the adverse immunological effects linked to combination phage therapy. Furthermore, covalent conjugation of diphtheria toxoid (DT) to PA-OMVs has shown exceptional protective efficacy in a burn wound infection model, greatly decreasing inflammatory cell infiltration and tissue damage [133]. Additionally, researchers developed a novel vaccine candidate—Pa-STING CNP—by encapsulating STING-adjuvanted nanoparticle cores within OMVs derived from highly virulent clinical isolates of *P. aeruginosa*. This vaccine induces robust anti-*P. aeruginosa* IgG antibody responses at extremely low doses (as low as 0.01 μg) and significantly enhances the recruitment and activation of APCs in lymph nodes, demonstrating excellent immunogenicity and potential protective efficacy [134].

To provide a critical comparison of the major therapeutic targets discussed in this review, we have summarized their key features—including supporting evidence, limitations, and translational readiness—in Table 3.

### Key Bottlenecks in the Clinical Translation of OMVs

Vaccines and treatments are the two main areas of clinical translation of OMVs. However, the development of OMV-based vaccines has progressed relatively slowly. Aside from the commercially available 4CMenB vaccine [10], most candidate vaccines remain in preclinical stages, with only a handful having advanced to clinical trials. Several key challenges must be addressed to expedite clinical translation: enhancing immunogenicity, improving yield, ensuring safety, and maintaining long-term stability.

To enhance immunogenicity, several strategies have been developed to efficiently display antigens on the OMV surface, thereby eliciting stronger protective immune responses. For instance, antigens can be displayed through fusion systems such as cytolysin A and adhesin involved in diffuse adherence [138, 139], or via conjugation methods [140]. However, high expression of fusion proteins may exert toxicity effects on bacterial hosts [141], limiting their application. Furthermore, multiple fusion systems must be screened for different target antigens to identify the most suitable approach for large-scale production. It is also critical to ensure that surface-displayed antigens maintain their native conformation and correct folding to effectively induce specific adaptive immune responses.

Under normal culture conditions, Gram-negative bacteria usually produce minimal amounts of OMVs. Studies indicate that increasing oxygen saturation [142], iron deficiency [143], or adding antibiotics to the medium [144] can all enhance OMVs production. Large-scale synthesis is facilitated by bioreactors, which provide improved control over oxygen levels and other parameters. However, scaled-up cultivation may introduce larger amounts of undesirable contaminants, such as cytoplasmic proteins [145]. Therefore, developing innovative manufacturing techniques for high-yield, high-purity OMV production remains a pressing challenge.

Safety is another major obstacle. Like other pharmaceuticals, OMV-based products must meet stringent safety and quality standards, yet unified standards are currently lacking. LPS, particularly its Lipid A moiety, while effectively inducing immune responses, may also trigger uncontrolled inflammatory reactions such as sepsis [146]. Currently, while LPS toxicity can be reduced through detergent treatment or genetic modification of LPS [48], detergent treatment may result in the loss of useful surface antigens on OMVs. Moreover, there are substantial technical challenges in creating LPS knockout strains. Together, these elements present significant obstacles to safety improvement.

Finally, long-term stability is also critical for the clinical application of OMVs. Ensuring that the product remains safe and effective for the duration of its designated shelf life is a key challenge. Over time, a decrease in product efficacy may result from improper storage conditions that jeopardize the vesicles' structural integrity. This undoubtedly poses a significant challenge when scaling up production to meet clinical demands.

Despite these challenges, recent studies have shown that X-ray-treated PA-OMVs can significantly suppress inflammatory cell infiltration into the lungs and reduce pro-inflammatory cytokine production (such as IL-1β and TNF-α) under both prophylactic and therapeutic conditions. This markedly alleviates lung injury pathology, offering a potential solution to OMV-based infectious disease therapy [147].

## Conclusion

*P. aeruginosa* is a highly virulent opportunistic pathogen capable of causing life-threatening acute and chronic infections, particularly in immunocompromised individuals with conditions such as bronchiectasis and cystic fibrosis. Antibiotic resistance in *P. aeruginosa* and the rising incidence of lethal infections have raised significant public health concerns, underscoring the urgent need to elucidate its pathogenic mechanisms and identify possible therapeutic targets. PA-OMVs have attracted considerable attention due to their immunogenic properties and critical role in pathogenesis. Clarifying the pathogenic pathways and possible targets of PA-OMVs is critical for guiding future research.

On the one hand, PA-OMVs serve as efficient delivery vehicles for virulence factors, facilitating pathogenic processes—including host immune evasion and biofilm formation—while exacerbating inflammatory cascades and contributing to tissue damage. On the other hand, PA-OMVs carry PAMPs, stimulating both innate and adaptive immune responses in the host. The immunoprotective properties of PA-OMVs have been conclusively demonstrated in extensive preclinical studies across multiple animal models, making them highly promising candidates for vaccine development and anti-infective treatments.

The “double-edged sword” nature of OMVs presents a major conundrum: how to maximize their immunostimulatory benefits while minimizing their toxicity. A crucial objective is striking a balance to achieve therapeutic benefit. Furthermore, little is known about the fundamental processes underpinning OMV biogenesis and host-pathogen interactions, or whether additional, unidentified mechanisms are involved. Moreover, a substantial translational gap remains. Although many *in vivo* studies with animal models show promise, clinical data in humans are scarce. Future research should focus on the following areas: developing cost-effective and scalable methods for OMV production and purification; applying synthetic biology to enhance OMV immunogenicity; exploring the synergistic effects of OMVs in combination therapies (*e.g.*, with antibiotics, phages, or immune checkpoint inhibitors); and attenuating the toxicity of OMV-associated virulence factors. Ultimately, the integration of emerging technologies positions engineered PA-OMVs as a promising option for addressing the growing antibiotic resistance crisis, and could revolutionize our approach to managing challenging bacterial pathogens. Greater attention should be paid to the potential applications of engineered PA-OMVs in the prevention and treatment of *P. aeruginosa* infections.

## Figures and Tables

**Table 1:** The main components of the porins of *P. aeruginosa* and their physiological role.

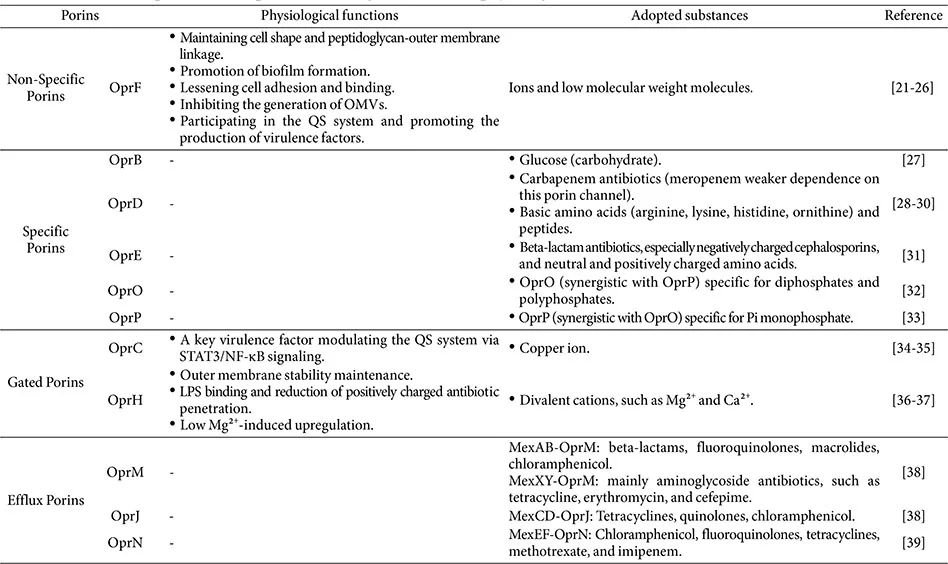

**Table 2:** Summary of major bacterial pathogen-associated molecular patterns.

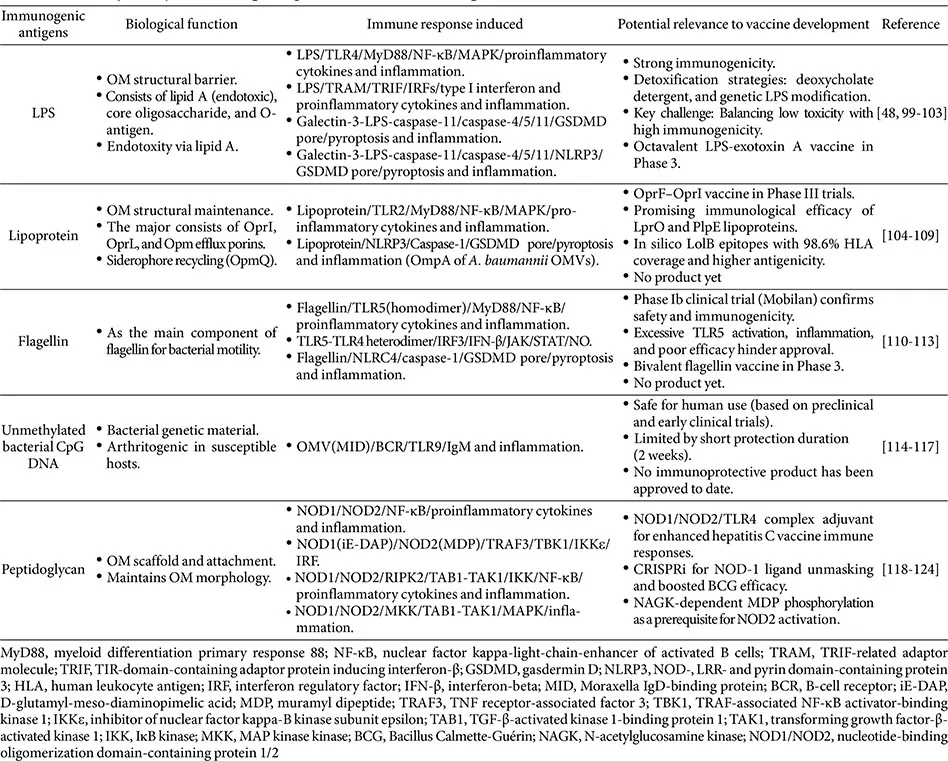

**Table 3:** Summary of therapeutic targets derived from PA-OMVs.